# Strategies to identify candidate repurposable drugs: COVID-19 treatment as a case example

**DOI:** 10.1038/s41398-021-01724-w

**Published:** 2021-11-16

**Authors:** Ali S. Imami, Robert E. McCullumsmith, Sinead M. O’Donovan

**Affiliations:** 1 Department of Neurosciences, University of Toledo, Toledo, OH USA; 2 Neurosciences Institute, Promedica, Toledo, OH USA

**Keywords:** Pharmacology, Molecular neuroscience

## Abstract

Drug repurposing is an invaluable strategy to identify new uses for existing drug therapies that overcome many of the time and financial costs associated with novel drug development. The COVID-19 pandemic has driven an unprecedented surge in the development and use of bioinformatic tools to identify candidate repurposable drugs. Using COVID-19 as a case study, we discuss examples of machine-learning and signature-based approaches that have been adapted to rapidly identify candidate drugs. The Library of Integrated Network-based Signatures (LINCS) and Connectivity Map (CMap) are commonly used repositories and have the advantage of being amenable to use by scientists with limited bioinformatic training. Next, we discuss how these recent advances in bioinformatic drug repurposing approaches might be adapted to identify repurposable drugs for CNS disorders. As the development of novel therapies that successfully target the cause of neuropsychiatric and neurological disorders has stalled, there is a pressing need for innovative strategies to treat these complex brain disorders. Bioinformatic approaches to identify repurposable drugs provide an exciting avenue of research that offer promise for improved treatments for CNS disorders.

## Introduction

**Fig. 1:**
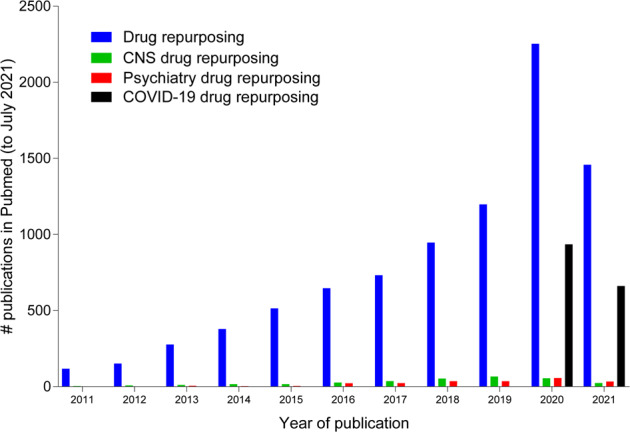
The number of publications referencing “drug repurposing” has increased year-on-year over the last decade. Psychiatry and CNS drug repurposing reflect a small proportion of total publications. By comparison, in under two years, COVID-19 related drug repurposing efforts have resulted in the generation of a large number of publications.

**Fig. 2:**
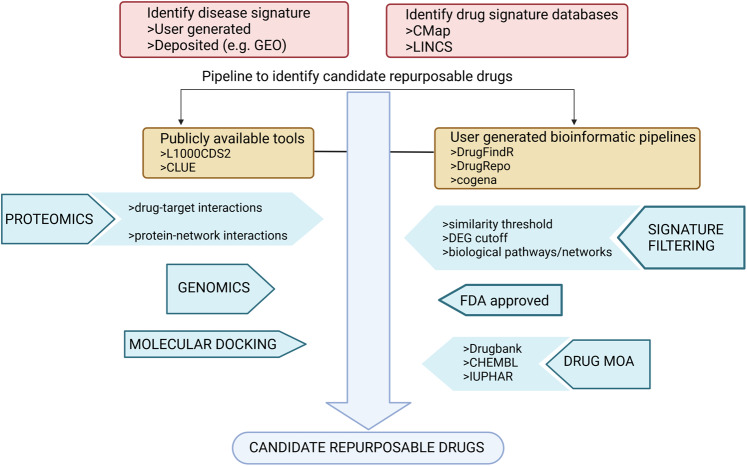
Bioinformatic drug repurposing workflows. Following selection of transcriptomic disease data and drug signature databases, a large number of publicly available or user-designed tools can be adapted to identify candidate repurpose drugs. Signatures are generated based on different criteria, including differential gene expression (DEG) cutoff and disease x candidate drug similarity score. Genomic and proteomic data can also be integrated into the workflow to provide insight into drug-target interactions, for example. Candidate repurposable drugs may be further filtered by relevance of drug mechanism of action (MOA), using molecular docking to determine how a drug interacts with a target protein, or to those drugs that are already FDA-approved. Created with BioRender.com.

The outbreak of the COVID-19 pandemic led to an unprecedented response in the scientific community to rapidly identify, develop and implement pharmacotherapies for the treatment of COVID-19 and its causative agent, the novel *coronaviridae* strain severe acute respiratory syndrome coronavirus 2 (SARS-CoV-2). In less than two years, over 6 000 clinical trials related to COVID-19 have been registered (clinicaltrials.gov) and almost 1 500 articles specifically related to drug repurposing for COVID-19 treatment can be found on PubMed (Fig. 1). Despite the massive investment of resources and a focused global effort, only a single drug, the broad spectrum antiviral remdesivir, is currently approved by the FDA for the treatment of COVID-19 [1]. This highlights the significant challenges to identifying pharmacotherapies for complex, heterogeneous diseases and is a familiar scenario to researchers in the field of neuropsychopharmacology. Although the initial urgency to identify drug treatments has waned with the development of effective vaccines, the emergence of new SARS-CoV-2 variants “of concern,” geographical inequities in vaccine availability and the predicted need for periodic booster shots ensure that effective drug treatments for COVID-19 are still an important tool to overcoming the pandemic. Here, we evaluate in silico approaches deployed to identify candidate repurposable drugs for the treatment of COVID-19 to determine what lessons can be gleaned from the accelerated and concerted efforts during the pandemic, and whether they can inform shortcomings in the identification of pharmacotherapies for CNS disorders. Using COVID-19 as a case study we will (1) summarize in silico methodologies applied to identify repurposable drugs (Fig. 2) and (2) discuss how they might inform current drug repurposing efforts for neuropsychiatric disorders. Selected representative studies will be discussed that highlight how different drug discovery approaches may be adapted by neuroscience researchers to address the pressing need for improved treatments for psychiatric conditions.

## COVID-19 case study

The focus of drug discovery efforts for COVID-19 has rested on drug repurposing or repositioning, that is, identifying novel uses for existing (approved and investigational) drugs that are different from their original indication [2]. The advantages of this approach include significant savings in terms of time and cost. Drug repurposing circumvents some of the safety, drug tolerability and dosage concerns associated with developing a drug lead from bench to bedside, shaving years off the drug development pipeline [3].

Within weeks to months of the COVID-19 pandemic, genomic, transcriptomic, and proteomic profiles were available for the causative SARS-CoV-2 virus and from infected human tissues. Data sharing was prioritized by institutions around the world and a surge of research papers soon followed. In silico approaches were deployed to mine the available data without the need for laboratory wet work in an era of quarantine and remote working. Established bioinformatic workflows were quickly adapted and applied to identify therapeutic targets and candidate repurposable drugs for the treatment of COVID-19.

## Machine learning approaches

The advancement in computer science and data storage have brought forth what could be easily termed as the “Age of Artificial Intelligence (AI).” “Deep Learning,” one of the methods to achieve AI in a particular system, has become the de-facto standard and is often used synonymously with AI. The field of biomedical research is not an exception to this trend. Over the past decade, numerous attempts have been made to integrate AI systems into diagnostics, epidemiology, outcome prediction and drug discovery [4–6]. There has been mixed levels of success in these fields and there is still a long way to go before AI can be used autonomously for drug discovery. However, at present, Deep Learning and AI allows for the rapid synthesis of heterogeneous data sources to narrow down lists of potential compounds of therapeutic value [4–8]. AI-based approaches have three major components, all subject to their own constraints and limitations. First and foremost is data availability. Second is the processing of the data sources and selection and application of a particular AI technique. Finally, there is the dual problem of results interpretation and predictive validity.

### Data Sources

Any machine learning (ML) approach requires high quality input data. This data will not only underlie the inferences that the AI/ML approach will generate but will also dictate the nature of the approach that is used [9]. Thus, it is an absolute necessity to generate databases that are easily accessible. Currently, there are several databases available online that encompass a variety of “omics.” These include primary databases like the National Center for Biotechnology Information’s (NCBIs), Gene Expression Omnibus (GEO; https://www.ncbi.nlm.nih.gov/geo/), PubChem (https://pubchem.ncbi.nlm.nih.gov/) and the European Bioinformatics Institute’s ChEMBL (https://www.ebi.ac.uk/chembl/) as well as secondary databases such as ExCape-DB [10] and PhIN [11] that curate data from literature and primary databases creating an inferential layer of data [12]. In addition, there are other data sources that generate predictions based on existing datasets. These can include predicted protein-protein [13] and drug-receptor [11] interactions. Finally, other resources include databases like DrugR + [14] where empirically defined and predicted interactions between drugs and their possible replacements (candidate repurposable drugs) has been curated. In the wake of the SARS-CoV-2 pandemic, several of these data sources were utilized to identify novel drugs and to repurpose existing drugs. Chief among these were the Library of Interconnected Network-based Cellular Signatures (LINCS) [15–17], DrugBank [18–20] and Protein Databank [21–23].

### Data processing

Once high-quality data sources have been identified, the next step is to integrate and process the data. For this purpose, there are several approaches that can be relied upon, including Logistic Regression, Support Vector Machines, and Convolutional and Recurrent Neural Networks [9]. Selection of a particular model is guided by the availability of appropriate datasets, the nature of the data itself and the expertize of the team conducting the analysis. In particular, the Convolutional Neural Networks have become the de-facto standard for deep learning in recent years due to their ability to learn necessary parameters from the data autonomously. The ML models rely on specific parameters that are unique to each combination of dataset, method, and problem. These parameters are usually only significant in a mathematical sense and not interpretable by humans in a meaningful way. In contrast, “hyperparameter tuning” selects the values for the parameters that influence how the learning algorithm works. It is a time-consuming step in the process of building ML/AI based models, but it is essential to achieve optimal setup of these “hyperparameters” to generate models that can provide more accurate results without sacrificing time or computational efficiency. It is evident that AI/ML has matured significantly over the past decade [24, 25]. With projects like Virtual Physiological Human [26, 27] on the horizon, which aim to provide a functioning in silico simulation of a human that can react to stimuli, we may look forward to the transition of more in silico identified drugs to clinical trials soon [28].

For identifying SARS-CoV-2 treatments, deep learning approaches that relied on convolutional neural networks and that integrated a varied type of information, including transcriptomics, drug structure and protein sequences, appeared to work the best [29]. Other approaches relied on molecular docking simulations in addition to the above-mentioned datasets [30, 31]. As early as February 2020, machine learning and AI driven approaches showed promise in identifying candidate repurposable drugs for COVID-19. Using BenevolentAI, the numb-associated kinase (NAK) family was identified as a drug target for the treatment of COVID-19. The NAK inhibitor Barcitinib has a high affinity for AP2 associated protein kinase 1 (AAK-1) and was identified as the most promising candidate drug from this AI screen. Inhibition of AAK-1, a regulator of clathrin-mediated endocytosis, was expected to inhibit SARS-CoV-2 infection of host cells [32, 33]. Clinical trial (NCT04401579) of combination therapy remdesivir and barcitinib found fewer side effects and greater efficacy in reducing COVID-19 patient recovery time than with remdesivir treatment alone [34]. The corticosteroid dexamethasone was also identified by different groups using AI approaches, like that applied by AI VIVO and [35], resulting in identification of a therapy that reduced mortality in ventilated COVID-19 patients in clinical trial [36]. These studies highlight the utility of in silico and AI/ML based approaches to successfully identify high yield repurposable drugs.

## Signature-based approaches

A gene signature describes the unique pattern of gene expression in a cell associated with biological or pathological (disease) processes or in response to genetic or pharmacological perturbation. Highly similar signatures might then represent novel connections between a drug and protein target, or similar function of two structurally dissimilar chemical perturbagens. Conversely, if drug and disease signatures are dissimilar, then that drug may act to reverse the disease phenotype (signature reversion principle) [37]. Disease-related transcriptomic data is most often user-generated and made available through public repositories like NCBI GEO https://www.ncbi.nlm.nih.gov/geo/). The need for a systematically generated catalog of cellular signatures led to the creation of LINCS [38], a product of a multi-institutional consortium. The LINCS platform (LINCS data portal 3.0) currently contains data on over 30,000 small molecules (http://lincsportal.ccs.miami.edu/SmallMolecules/) and over 2 million different chemical perturbagen transcriptomic signatures. It also provides a large range of tools for exploration and analysis of data. The L1000 is an assay of 978 landmark genes that provides a reduced representation (approximately 82%) of the transcriptome [39] and facilitated the systematic generation of gene signatures for almost 20,000 small molecules. The catalog of signature connections is the connectivity map (CMap) [39, 40]. Tools for analysis of the massive L1000 datasets include the L1000 characteristic direction signature search engine (L1000CDS2) [41], iLINCS, a web platform for analysis of LINCS transcript and proteomic data and signatures [38], the Connectivity map (CMap), and LINCS Unified Environment (CLUE; https://clue.io). CLUE is a cloud-based query infrastructure that allows researchers to explore and seamlessly incorporate CMap data into their analyses. In addition, LINCS signature data is easily downloaded and integrated into user-designed pipelines to fit their experimental needs. In the hands of individual users, the signature data provided by the LINCS resources has generated hundreds of candidate repurposable drugs for the treatment of COVID-19 [17, 42–44]. An advantage of the LINCS and CMap platforms is their accessibility. With little or no formal bioinformatic training, the provided tools can be utilized by bench scientists to explore datasets of interest. With basic R skills, user-generated pipelines can be designed to interrogate LINCS datasets, as discussed below.

### Examples of signature-based approaches to identify COVID-19 candidate repurposable drugs

Although almost all COVID-19 signature-based analyses use the same or similar publicly available RNAseq data from SARS-CoV-2 infected cell-lines and infected host tissues and cells [45, 46], different bioinformatic pipelines have identified hundreds of candidate drugs, perhaps raising concerns about the specificity and utility of such approaches. However, although hundreds of unique candidate drugs have been proposed for repurposing, these drugs are often members of the same drug class. Unsurprisingly, antiviral drugs were often reported as top hits, however, other antimicrobials, immunosuppressants and kinase inhibitors were also commonly reported [35, 43, 47]. Kinase inhibitors were of growing interest as antivirals prior to COVID-19 [48, 49]. The identification of drugs with immunosuppressant properties as top candidates [17, 50], amid the emergence of the “cytokine storm” as a major treatment concern during COVID-19, highlights the value of the signature-based approach. Additionally, signature-based approaches can be adapted for more targeted search efforts, for example in the case of COVID-19, to find drugs that act on the ACE2 receptor [51].

Here, we describe how signature-based approaches, in particular using the LINCS signature database, can be adapted to identify candidate drugs using publicly available tools or user generated bioinformatic pipelines (and could be applied to other disease states). Islam et al identified a set of hub genes from gene enrichment of SARS-CoV-2 transcriptome data and analysis of protein-protein interaction networks in lung tissue, for drug repositioning using LINCS [42]. Hub genes from the protein-protein interaction networks, generated using DifferentialNet Database [52] and NetworkAnalyst [52] tool and with a degree filter (>30), were considered to represent essential biological signaling molecules. The top 10 hub proteins were used for drug repurposing. Using the L1000CDS2 search engine, the chemical perturbagens with the lowest cosine similarity score for the input targets were identified. The top 10 chemical perturbagens were carried forward for further consideration; drug mechanism of action was checked in publicly available databases (e.g., Drugbank, IUPHAR) and the literature to ensure specificity. Of the top 10, kinase inhibitors and antimicrobials were represented, with 7 drugs having approval status and the remaining 3 having investigational status. Molecular docking analyses was carried out to further validate the bioinformatically determined candidate drugs.

Others develop bioinformatic pipelines to identify and curate candidate drugs on specific criteria deemed important for drug consideration. For example, using the R shiny application DrugFindR, our group extracted L1000 genes from publicly available COVID-19 transcriptome datasets. Candidate repurposable drugs were identified if 1) they had chemical perturbagen signatures in at least 5/7 common cell lines in LINCS, 2) if the chemical perturbagen signature had a minimum discordance score of <−0.321 with COVID-19 disease signatures and 3) a concordance score >0.321 with known antivirals in use for COVID-19 treatment at that time and 4) if candidate drugs were FDA-approved [17]. Following manual curation of the literature and candidate drug list, a final “top hit” list was generated containing drugs that were already undergoing clinical trial for COVID-19, confirmed to specifically inhibit SARS-CoV-2 in vitro or had known *coronavirus* antiviral effects. Using a modified DrugFindR workflow, the potential mechanisms of action of two candidate drugs for the treatment of COVID-19, oxytocin [53] and fluoxetine [54] were also explored. Analysis of LINCS consensus gene knockdown signatures found that the oxytocin gene signature is highly concordant with inflammation knockdown signatures (e.g., IL-6 knockdown) but discordant with signatures of pro-immune marker knockdown, like CD40. Signature analysis also provided support for an anti-inflammatory role for the antidepressant fluoxetine, via the NF-kappB-IL6-ST signal transduction pathway. Fluoxetine (NCT04377308) is currently undergoing clinical trial for COVID-19 and is expected to treat the cytokine storm associated with disease.

Alternative LINCS signature-based workflows include the DrugRepo pipeline (https://nelhachem.shinyapps.io/DrugRepo/?_ga=2.78354771.679352558.1592320962-118407775.1587897066) and [55]. This pipeline integrated LINCS chemical perturbagen gene signature data with gene and drug set enrichment analysis and drug-target associations to identify candidate repurposable drugs [55]. The authors curated drug-target interactions from DrugBank [18, 19], IUPHAR [56] and CLUE (https://clue.io). Drug set enrichment, which uses drug-target associations instead of gene sets, enriched for drug-target interactions among the identified candidate drugs. As an additional filter, protein targets with fewer than 3 representative drugs were removed. Importantly, analyzing a ruxolitinib-treated A549 cell line infected with SARS-CoV-2 (SARS2_A549_ACE2_RUXO; [45]) using this pipeline resulted in identification of ruxolitinib as a top hit. Drug-set enrichment analysis then confirmed that drugs of the ruxolitinib drug class, JAK2 inhibitors, were also overrepresented in their results, validating their approach and confirming the accuracy of the L1000 drug identification.

The co-expressed gene set enrichment analysis (cogena) is a framework that uses the CMap drug induced gene signatures to identify drugs that reverse differentially expressed and co-regulated genes [57]. Coexpressed genes are often expressed in the same biological pathways and are hypothesized as more likely drivers of the underlying disease biology, adding an extra dimension to selecting gene sets for targeting drug repositioning. This approach identified several drugs including two approved antiviral drugs (saquinavir and ribavirin) that were previously implicated by molecular docking approaches for the treatment of COVID-19 [44].

Signature-based analyses are most often based on gene expression (transcriptome) data, however, integrating protein-network data can add another dimension to analyses. Zhou et al devised a drug-target network of human coronavirus (HCoV)-host protein interactions. A network of 119 proteins that were targets of HCoV (6 different strains) or in pathways essential for infection by human coronaviruses were identified [58]. The drug-target network was compiled from drug-target interaction data from multiple sources including DrugBank [18, 19] and ChEMBL [59]. Drug-target interaction criteria included binding affinity (EC50 ≤ 10 µM) and inclusion of a unique, reviewed accession number for that protein in UniProt. Druggable proteins were then mapped into the HCoV-host interactome where the authors found almost 40% of the protein interactome was targeted by at least one (approved or under clinical trial) drug. To capitalize on the benefits of a network-based targeting approach [60–63], the human protein-protein interactome was expanded and a network proximity measure was applied to quantify connections in the HCoV-specific subnetwork, resulting in a larger list of 135 candidate repurposable drugs. Additional criteria, including network proximity score, enrichment of GSEA gene signatures from HCoV transcriptome datasets, drug CMAP signature, and literature evidence of drug antiviral efficacy and side-effect profile, resulted in 16 candidate drugs, including anti-inflammatory, immunosuppressant and antineoplastic agents, estrogen receptor modulators and angiotensin receptor blockers. This experimentally validated drug-target network approach was readily adapted by the group in response to the COVID-19 outbreak [60–62], has multiple levels of validation for candidate drug identification and incorporates transcriptome validation as well as a protein-network focus. Drug repurposing efforts that integrate multi-omic signatures with structure data [64] and machine learning approaches have successfully identified drug candidates with anti-SARS-CoV-2 activity in vitro [65].

Overall, signature analysis using CMap, CLUE and LINCs datasets and user-developed pipelines have been adapted by many different groups to identify hundreds of potential candidate repurposable drugs [66–68]. By prioritizing gene input for disease signature generation based on pathway analyses and gene-set enrichment analyses from infected tissues or allowing virus-host and drug-target associations (DrugBank, ChemBL, IUPHAR) to inform the selection of drugs, signature-based approaches can be adapted for targeted (e.g., specific drug class or biological pathway), biologically relevant candidate repurposable drug identification. Applying different filtering criteria (signature similarity scores, FDA approval, drug mechanism of action, manual curation of drugs in clinical trial) can also improve stringency of candidate drug identification.

It should be noted that while a number of candidate repurposable drugs show promise as antivirals in vitro [43, 69, 70] and many more are under investigation in clinical trial, safety concerns [71–73] and false positive findings [74] are common upon deeper exploration. Results from the RECOVERY (https://www.recoverytrial.net/results) and SOLIDARITY [75] platform clinical trials of drugs that showed early promise, like hydroxychloroquine and lopinavir, have since proven to have little or no effect in reducing mortality rates or treating COVID-19 [76].

## Application to drug repurposing for CNS disorders

Even in the case of COVID-19, where we have knowledge of the causative pathogen and sufficient understanding of the molecular mechanisms of infection to identify the viral proteins that can be targeted to inhibit viral replication, development of approved treatments is a challenge. For CNS diseases, a major stumbling block to the identification of effective therapies remains our limited understanding of the etiology of these disorders. The heterogeneity and complexity of CNS disorders undoubtedly contributes to the very low rates of clinical approval of new drugs, which often fail in clinical trials due to a lack of efficacy [77]. For drugs that attempt to treat the causes of neurodegenerative diseases like Alzheimer’s disease and Parkinson’s disease, the clinical failure rate has been high [78]. The shortcomings of animal models [79] and difficulties in designing drugs that have the physiochemical properties to be successfully delivered across the blood brain barrier are other limiting factors [80]. The challenges to successful drug discovery and drug repurposing in the CNS have been reviewed in detail elsewhere [78, 81–84].

A standard approach to discover new drugs, high-throughput screening to identify compounds that show activity against a single target or pathway, has resulted in diminishing success over the years [85], and has been particularly limited for brain-related disorders. For example, for Alzheimer’s disease the “amyloid hypothesis” led to the identification of a target pathological pathway and a search for drugs that can inhibit beta-amyloid aggregation. This has not resulted in successful disease-modifying treatment [86]. Drug repositioning using signature-based and machine-learning approaches offers an alternative strategy for drug identification for brain-related disorders, which to date has primarily relied on serendipitous findings [87] and incremental improvements of existing drugs for treatment [78].

Signature-based strategies are already identifying promising drug leads for different brain disorders. Utilizing a combination of over 60,000 CMap and L1000 drug signatures, and transcriptomic and proteomic Alzheimer’s disease signatures, Lee et al devised a “drug repositioning perturbation score” to measure the inverse association between drug and disease signatures, weighted by pharmacogenomic knowledge, to identify candidate repurposable drugs with high score in both transcriptomic and proteomic data [88]. Top findings included inhibitors of monoamine oxidase, which are implicated in the pathology of Alzheimer’s disease [89]. This approach has the advantage of incorporating different “omics” data types to inform drug discovery. The CMap was also deployed to identify candidate repurposable drugs for bipolar disorder, using an approach that focused on similarity with current indicated drugs for bipolar disorder rather than discordance with disease signature. Common differentially expressed genes, identified by RNAseq analysis of models, a neuronal cell line (NT2-N), and rats treated with a combination of bipolar disorder drugs (lithium, valproic acid, quetiapine, lamotrigine), were used to generate the input drug gene signatures. CMap drug signatures of 10 drugs that were concordant with the signatures of known bipolar disorder drug treatments were identified [90]. As an alternative to “omics” to generate disease signatures, Sullivan et al experimentally identified enzymes associated with bioenergetic deficits in postmortem schizophrenia brain, followed by in silico confirmation of these target or “seed genes” in other postmortem datasets [91]. In iLINCs, gene knockdown signatures for each seed gene were generated. Next, drugs with gene signatures that were highly discordant to the seed gene signatures were identified, converging on PPAR agonists as candidate drugs of interest. Administering the PPAR-gamma agonist pioglitazone to GluN1 knockdown mice reversed cognitive deficits associated with this phenotype and was proposed as a target drug of interest for treatment of cognitive symptoms associated with schizophrenia and other neuropsychiatric conditions [91]. Taking a different approach to generating disease signatures, a multi-psychiatric disorder analysis generated signatures from genome wide association study (GWAS)-imputed transcriptome profiles [92]. GWAS-imputed transcriptome data captures the genetically regulated part of expression and overcomes some limitations of transcriptome-only derived signatures, including circumventing confounding factors like medication effects that are associated with signatures generated from postmortem tissue datasets. Applying drug-set enrichment analysis generally supported the validity of the resulting drug repurposing findings. For example, known antipsychotic drugs were enriched in schizophrenia and bipolar disorder analyses and known antidepressant were among the top findings for MDD. GWAS was also used to generate gene-set associations with disease phenotype (MDD and schizophrenia) prior to integration with drug-target relationships and chemical perturbagen signatures from CMAP [93, 94]. By constructing bipartite drug-target interaction networks from these analyses, this approach assesses whether identified drugs interact with multiple targets, and whether different targets show affinity for dissimilar drugs, allowing for prioritization of drug-gene sets for further exploration [95].

One of the major limiting factors when trying to understand the pathophysiology of psychiatric disorders and identify new therapeutics is disease heterogeneity. One approach taken to address this issue is exploring postmortem transcriptome signatures generated from subjects in depressive and remission stages of MDD and non-psychiatrically ill controls [96]. Gene co-expression analysis and Bayesian network modeling identified gene modules associated with disease state (vs trait) which was then searched against CMap signatures to identify candidate repurposable drugs. A subset of the drugs identified are already in use to treat psychiatric disorders. Such an approach can be used to identify treatments that can be targeted to clinical phases of MDD.

An advantage of signature-based approaches is the flexibility to incorporate emerging “omics” analyses, which will improve our understanding of disease mechanisms. Single-cell RNAseq studies offer significant insight into complex tissues like human brain, identifying altered patterns of cell-type specific gene expression in cell populations that are associated with neurological [97–99] and psychiatric [100] disorders. Similarly, transcriptome analysis of neuronal and glial iPSCs and cerebral organoids [101–104] derived from patients with neurological and psychiatric disorders is a useful tool, with some translational potential for interrogating disease mechanisms [105] and can be incorporated into signature-based drug repurposing analyses [106]. Although still limited relative to the available transcriptomic signatures, drug signatures for the P100, the LINCS proteomic assay platform consisting of approximately 100 representative phosphopeptide probes analyzed by mass spectrometry, offers another strand of data for drug signature identification.

A number of logistical hurdles should also be considered prior to conducting in silico drug repurposing. Many of the analyses reviewed here require standard computing resources found in most laboratories. However, machine learning dependent approaches may require access to a computer cluster, to provide sufficient processing power. Additionally, the databases referenced here, including LINCS, PubChem, GEO, IUPHAR etc. are publicly accessible at no cost to the user, and provide convenient interfaces for scientists to conduct analyses. However, some databases, like DrugBank, may require the creation of an account and verification of academic status prior to accessing their complete database.

Finally, limiting candidate repurposable drugs from in silico screens to those that have obtained FDA approval and/or are under investigation addresses a major concern in drug development, namely, drug safety. However, further assessment of any candidate drug is essential to determine if the drug is efficacious for its purported novel use, within its established safe dosage range. In addition, a vast array of in silico toxicology resources are available to assess drug toxicity during the drug development process. In a recent review, Pawar et al. identified almost 1,000 data resources including chemical information, and pharmacovigilance and toxicity data that could inform on toxicology during drug discovery [107]. Projects like the “Enhancing TRANslational SAFEty Assessment through Integrative Knowledge Management (eTRANSAFE)” (https://etransafe.eu/) are developing the infrastructure to integrate analyses of different data types to improve translational safety assessment during drug development. Integrating computational toxicology and drug repurposing approaches will undoubtedly improve the transition of candidate repurposable drugs to the clinic, by identifying potential safety concerns earlier in the process.

In summary, a large number of bioinformatic workflows and pipelines have been developed in the last two years to address the urgent need to identify candidate repurposable drugs for COVID-19. Many of these workflows may now be adapted to drug repurposing efforts for neurological and neuropsychiatric disorders, where drug discovery efforts have stagnated. Signature-based approaches using available CMap and LINCs tools are particularly amenable to use by those without formal bioinformatic training. Although significant challenges remain to successfully translate candidate drugs from bench to bedside, advances in bioinformatic drug screening may open new avenues for identifying therapies for CNS disorders.
